# Serum Oxidative Stress Markers and Genotoxic Profile Induced by Chemotherapy in Patients with Breast Cancer: A Pilot Study

**DOI:** 10.1155/2015/212964

**Published:** 2015-10-20

**Authors:** Antonio Luiz Gomes Júnior, Marcia Fernanda Correia Jardim Paz, Laís Iasmin Soares da Silva, Simone da Costa e Silva Carvalho, André Luiz Pinho Sobral, Kátia da Conceição Machado, Paulo Michel Pinheiro Ferreira, Prabodh Satyal, Rivelilson Mendes de Freitas, Ana Amélia de Carvalho Melo Cavalcante

**Affiliations:** ^1^Laboratory of Genetic Toxicity, Postgraduate Program in Pharmaceutical Sciences, Federal University of Piauí, 64049-550 Teresina, PI, Brazil; ^2^Postgraduate Program in Genetics, State University of São Paulo, 14049-900 Ribeirão Preto, SP, Brazil; ^3^Laboratory of Experimental Cancerology, Department of Biophysics and Physiology, Postgraduate Program in Pharmaceutical Sciences, Federal University of Piauí, 64049-550 Teresina, PI, Brazil; ^4^Chemistry Department, University of Alabama in Huntsville, Huntsville, AL 35899, USA; ^5^Laboratory of Research in Experimental Neurochemistry, Postgraduate Program in Pharmaceutical Sciences, Federal University of Piauí, 64049-550 Teresina, PI, Brazil

## Abstract

The aim of this study was to evaluate the oxidative parameters of erythrocytes and genotoxicity in leukocytes of patients with breast cancer. Oxidative parameters were detected by spectrophotometry and genotoxic damage by single cell gel electrophoresis. Twenty-eight women with breast cancer were monitored before chemotherapy and after the second and fourth cycles of therapy with cyclophosphamide and doxorubicin. After the fourth cycle, increases (*P* < 0.05) in the reactive substances to thiobarbituric acid levels, nitrite content, and superoxide dismutase activity and high rates of DNA damage in leukocytes were observed when compared with healthy women group and baseline levels. Similarly, after the second cycle, the same parameters were increased (*P* < 0.05) when compared with baseline levels. Increase in catalase activity was detected only after the fourth cycle and reduced glutathione levels and glutathione peroxidase activity were decreased in all cycles when compared with healthy women, as well as after the second and fourth chemotherapy cycles compared to baseline (*P* < 0.05). Patients with breast cancer presented an indicative of oxidative stress before, during, and after chemotherapy, as well as increased genotoxic damage in all stages of treatment, demonstrating the clinical applicability of this investigation.

## 1. Introduction

The etiology of breast cancer possesses a multifactorial origin [1, 2], showing as risk factors reproductive age, early menarche, late menopause, nulliparity, exogenous hormones, smoking, obesity, diet, alcohol consumption, physical inactivity, and genetic and environmental factors [1–5].

Most chemotherapeutic agents are not specific against neoplastic cells, also affecting normal cells [6], which results in a wide range of adverse reactions in virtually all tissues of body such as bone marrow suppression, alopecia, fatigue, generalized rash, diarrhea, and dizziness [7, 8]. Cyclophosphamide, one of the most used anticancer compounds, is a bifunctional alkylating member of the nitrogen mustard family that induces various types of DNA damage, such as DNA adducts, gene mutations, and chromosomal aberrations [6, 9]. In clinical and trials protocols, cyclophosphamide is used in combination with doxorubicin, an anthracycline agent capable of intercalating into DNA [10]. Their mechanism of cytotoxicity includes intracellular production of free radicals, DNA intercalation, and subsequent inhibition of DNA topoisomerase II [6, 9, 10].

Reactive oxygen species (ROS) represent important factor in carcinogenesis and may play a role in initiation and progression of tumors. Free radicals stimulate oxidative DNA damage, contributing to mutagenesis, which is essential for the process of tumor initiation [11–13]. Unrepaired DNA damage has been associated with a variety of human disorders including cancer and neurodegenerative diseases. When DNA is properly repaired, the injuries are inactivated and the cells return to normal cell cycle operation. If this damage is not repaired, specific cellular responses such as cell death, senescence, or uncontrolled proliferation could result. This damage may consist of small lesions in very specific sites within the DNA molecule, as adducts, cross-links, abasic sites, and points of gross abnormalities [14, 15]. The extent of this damage caused by ROS can be maximized or minimized by enzymatic (catalase, superoxide dismutase, and glutathione peroxidase) or nonenzymatic (vitamins A, C, and E, selenium, and reduced glutathione (GSH)) [2, 16–18] mechanisms of antioxidant defense. Based on this approach, the present study evaluated the antioxidant and genotoxic profile in blood cells of patients receiving a combined chemotherapy of adriamycin (doxorubicin) and cyclophosphamide (AC).

## 2. Materials and Methods

### 2.1. Study Population and Sample Collection

The subjects were patients diagnosed with ductal breast cancer under treatment at the Department of Oncology, São Marcos Hospital, Teresina, Piauí, Brazil, from August 2012 to February 2013. This clinical study was approved by the Research Ethics Committee of University Center UNINOVAFAPI (registration number 0406.0.043.00011). This study involved a total of 56 individuals including 28 patients exposed to chemotherapy by the AC protocol (adriamycin 60 mg/m^2^ and cyclophosphamide 600 mg/m^2^) and 28 patients not exposed to the chemotherapy. The patients were exposed to four 21-day cycles with intravenous AC. The unexposed group consisted of individuals who had not been exposed to genotoxic agents (including radiation and chemicals) and who were free of any malignant neoplasm or clinical, biochemical, hematological, hepatic, cardiovascular, renal, or endocrine manifestations. Blood samples were collected with EDTA or heparin by venipuncture using vacutainers, maintained at 4°C during transport to the laboratory, and immediately processed. Three collections of the peripheral blood were carried out during four cycles of chemotherapy: the first collection was performed before the beginning of treatment (C0), 21 days after the second cycle of chemotherapy (C2), and 21 days after the fourth cycle (C4).

All individuals in this study were submitted to a questionnaire from International Commission for Protection against Environmental Mutagens and Carcinogens [19], which included questions regarding standard demographic data (e.g., age and gender), medical issues (e.g., exposure to X-rays, vaccinations, and medications), lifestyle (e.g., smoking, coffee and alcohol consumption, diet, etc.) and occupation, such as number of working hours per day and protective measures adopted (PPE). In all groups, individuals who smoked more than 20 cigarettes per day were considered smokers [20].

After the questionnaires, data were analyzed using SPSS 17.0. (Chicago: SPSS Inc.) and the demographic, medical and lifestyle were summarized in Table 1.

Details about clinical features, such as cancer site, clinical stage and HER-2/neu, ER (estrogen receptor), and PR (progesterone receptor)* status*, were obtained and analyzed from medical records. The descriptive statistics for such variables are listed in Table 2.

### 2.2. Comet Assay

The alkaline comet (*single cell gel electrophoresis* (SCGE)) assay was performed as described by Singh et al. [21] with modifications suggested by Tice et al. [22]. Blood cells (5 *μ*L) were embedded in 95 *μ*L of 0.75% low-melting point agarose, which was immediately added to the surface of a precoated (1.5% agarose) microscope slide. When the agarose had solidified, the slides were placed in lysis buffer (2.5 M NaCl, 100 mM EDTA, and 10 mM Tris; pH 10.0–10.5) containing freshly added 1% (v/v) Triton X-100 and 10% (v/v) dimethyl sulfoxide (DMSO) for a minimum of 1 day and a maximum of 7 days. After treatment with lysis buffer, the slides were incubated in freshly made alkaline buffer (300 mM NaOH and 1 mM EDTA; pH N 13) for 20 min and the DNA was electrophoresed for 20 min at 25 V (0.90 V/cm) and 300 mA after which the buffer was neutralized with 0.4 M Tris (pH 7.5) and dried overnight. Gels were rehydrated for 5 min in distilled water and then stained for 15 min (37°C) with a solution containing the following sequence: 34 mL of Solution B (0.2% w/v ammonium nitrate, 0.2% w/v silver nitrate, 0.5% w/v tungstosilicic acid, 0.15% v/v formaldehyde, and 5% w/v sodium carbonate) and 66 mL of Solution A (5% sodium carbonate). The staining was stopped with 1% acetic acid and the gels were air dried. Analyses (100 cells/patient) were carried out by light microscopy (Olympus CX40) at 100x magnification with immersion oil. Images of cells (50 cells/slide in two replicates) were evaluated for the following: (i) damage index (DI), in which each cell was classified into classes (no damage = 0, maximum damage = 4) according to tail size and cell shape [23], with resulting values for each individual ranging from 0 (0 × 100) to 400 (4 × 100); (ii) damage frequency (DF), calculated as the percentage of injured cells. International guidelines and recommendations for the comet assay consider the visual scoring of comets to be a well-validated evaluation method. Although the DI parameter is often subjective, it has high correlation with computer-based image analysis [22, 24, 25].

### 2.3. Nitrite Content

The determination of nitrite content was based on the Griess reaction [26] in which 500 *μ*L of Griess reagent was added in white tube plus 500 *μ*L of distilled water (Blank) was added in white tube. In another test tube 500 *μ*L of Griess reagent and 500 *μ*L of the homogenate at 10% of the erythrocytes (sodium phosphate buffer 50 mM pH 7.4) (Test) were added. The spectrophotometric measurement was performed at 560 nm. Results were expressed in *μ*M/mg protein.

### 2.4. Thiobarbituric Acid Reactive Substances (TBARS) Levels

Blood samples were centrifuged at 3000 rpm at 4°C during 5 minutes. Plasma was removed and a pellet of erythrocytes was washed with a cold solution of NaCl 0.9% and centrifuged. An erythrocytes' homogenate 10% diluted in phosphate buffer sodium 50 mM and pH 7.4 was stored at −20°C. Lipid peroxidation was measured by TBARS levels, a method previously described by Draper and Hadley (1990) [27, 28]. 250 *μ*L of homogenate, 1 mL of trichloroacetic acid 10%, and 1 mL of thiobarbituric acid 0.67% were mixed and stirred. Subsequently, this mixture was maintained in a bath of boiling water for 15 min and freshened under running water. After cooling, 2 mL of n-butanol was added and centrifuged at 1.200 rpm/5 min and the butanol phase was read spectrophotometrically at 535 nm. Results were expressed as nmol/mL.

### 2.5. Reduced Glutathione (GSH) Levels

Determination of GSH was based on the Ellman reaction (5,5′-dithiobis-2-nitrobenzoic acid) with some modifications described by Sedlak and Lindsay [29]. Four hundred microliters of erythrocytes' homogenate (EDTA pH 5.4 buffer) was mixed with 320 *μ*L of distilled water and 80 *μ*L of trichloroacetic acid 50%. After centrifugation at 3.000 rpm for 15 min, 400 *μ*L from the supernatant was collected and added to 800 *μ*L of Tris-HCl 0.4 M, pH 8.9, and 20 *μ*L of DTNB 0.01 M. One minute later, spectrophotometric measurement was performed at 412 nm. Concentration of GSH was expressed in mg/g of hemoglobin.

### 2.6. Glutathione Peroxidase (GPx) Activity

The glutathione peroxidase activity coupled assay was determined by Paglia and Valentine [30]. GPx catalyzes the reduction of hydrogen peroxide (H_2_O_2_), oxidizing reduced glutathione (GSH) to form oxidized glutathione (GSSG). GSSG is then reduced by glutathione reductase (GR) and *β*-nicotinamide adenine dinucleotide phosphate (NADPH) forming NADP+ (resulting in decreased absorbance at 340 nm) and recycling the GSH. Because GPx is limiting, the decrease in absorbance at 340 nm is directly proportional to the GPx concentration. 1 unit of GPx-1 = the amount of enzyme necessary to catalyze the oxidation (by H_2_O_2_) of 1.0 *μ*mole GSH to GSSG, per minute at 25°C, pH 7.0. Results were expressed in U/g of hemoglobin.

### 2.7. Catalase (CAT) Activity

Erythrocytes' homogenate in pH 7.4 was centrifuged (800 g, 20 min) and the supernatant was used to quantify catalase activity. The reaction medium was prepared with H_2_O_2_ (18 mL), Tris HCl 1 M, EDTA pH 8.0 5 mM (1.0 mL), and H_2_O (0.8 mL). The reading was carried out in a quartz cuvette at 230 nm with 980 *μ*L of reaction medium plus 20 *μ*L erythrocytes' homogenate prepared in sodium phosphate buffer 50 mM, pH 7.4 [31].

### 2.8. Superoxide Dismutase (SOD) Activity

Erythrocytes homogenate prepared in sodium phosphate buffer 50 mM, pH 7.4, was centrifuged (800 g, 20 min) and supernatants were used for testing superoxide dismutase (SOD) activity. Cytochrome *c* reduction rate was determined by superoxide radicals using the xanthine-xanthine oxidase system as a source of superoxide anion (O_2_^−^) [32]. Results were expressed as U/mg protein. One unit (U) of SOD activity corresponds to the inhibition of 50% of O_2_^−^ in the presence of cytochrome *c*.

### 2.9. Statistical Analyses

In order to determine statistical differences, data expressed as mean ± standard error of the mean (S.E.M.) were compared by one-way analysis of variance (ANOVA) followed by the Newman-Keuls test (*P* < 0.05) using the Graphpad program (Intuitive Software for Science, San Diego, CA) and SPSS (version 19, SPSS Inc.). Correlations among data obtained were calculated using Spearman's correlation coefficient.

## 3. Results

### 3.1. Evaluation of Oxidative Stress

Evaluation of oxidative stress in patients with breast cancer in AC chemotherapy was performed by analyzing enzymatic and nonenzymatic parameters in erythrocytes by serum thiobarbituric acid reactive substances (TBARS) level, nitrite content, GSH concentration, and GPx, CAT, and SOD activities.

Results showed that the status of oxidative stress (*P* < 0.05) increased, as demonstrated by basal TBARS (1.42 ± 0.45 nM/mg of protein) and nitrite (1.16 ± 0.62 *μ*M/mg protein) contents in erythrocytes of patients with breast cancer when compared with the control group (0.37 ± 0.09 nM/mg of protein and 0.16 ± 0.05 *μ*M/mg of protein, resp.) (*P* < 0.05). When these same patients were submitted to chemotherapeutics (combination of cyclophosphamide and doxorubicin), such increases in both TBARS (4.76 ± 0.68 and 11.98 ± 0.65 nM/mg of protein) and nitrite ion levels (1.81 ± 0.02 and 3.49 ± 0.07 *μ*M/mg of protein) were also detected in C2 and C4, respectively (*P* < 0.05; Table 3).

Red blood cells of the patients revealed decrease in reduced glutathione concentration at 36.1% (24.94 ± 1.51 U/g protein) in comparison with control group (36.13 ± 7.65 U/g protein) (*P* < 0.05). With AC chemotherapy, there was decrease in GSH levels in C2 (46.6%) and C4 groups (50.9%) (*P* < 0.05). Similarly, baseline levels (C0) also presented diminution of 21.4% and 27.7% in C2 and C4 groups, respectively (Table 3).

**Figure 1:**
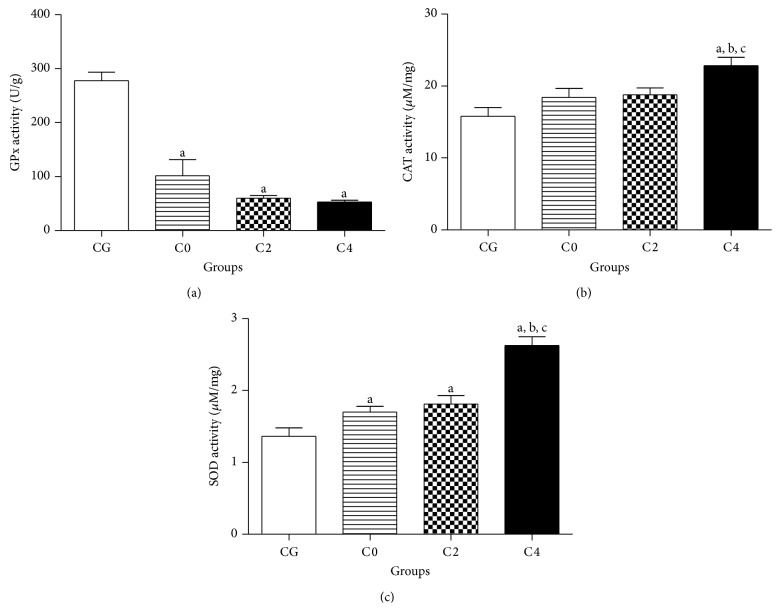
Antioxidant enzymes activity in erythrocytes of patients with breast cancer before (C0), during (C2), and after (C4) AC chemotherapy. Control group (CG) is represented by healthy patients. Values represent mean ± S.E.M. ^a^ *P* < 0.05 when compared with control group (CG) by ANOVA followed by *t-Student-Newman-Keuls*. ^b^ *P* < 0.05 when compared with C0 group (before chemotherapy) and ^c^ *P* < 0.05 when compared with C2 group (second cycle of chemotherapy).

GPx activity (Figure 1(a)) showed reduction (63.32, 78.31, and 81.0%) in erythrocytes of the patients with breast cancer activity in all groups analyzed (101.90 ± 29.48, 60.25 ± 4.66, and 52,77 ± 3.26 U/g for C0, C2, and C4, resp.) when compared to the control group (277.8 ± 15.88 U/g), respectively (*P* < 0.05).

In relation to the catalase levels, only treated patients (C4, 22.83 ± 1.17 *μ*M/mg) exhibited a significant increase (44.3%) when compared to the control group (15.82 ± 1.21 *μ*M/mg), C0, and C2 (18.44 ± 1.24 and 18.77 ± 0.96 *μ*M/mg, resp.) (Figure 1(b)). Similarly, superoxide dismutase activity (Figure 1(c)) also increased (33.1%) after the second cycle of chemotherapy (1.81 ± 0.63 *μ*M/mg) when compared with control group. After chemotherapy (2.63 ± 0.65 *μ*M/mg), its activity increased about 93.4 and 54.7% in relation to the control group (1.36 ± 0.62 *μ*M/mg) and baseline (1.70 ± 0.43 *μ*M/mg), respectively.

### 3.2. Index and Frequency of DNA Damage

**Figure 2:**
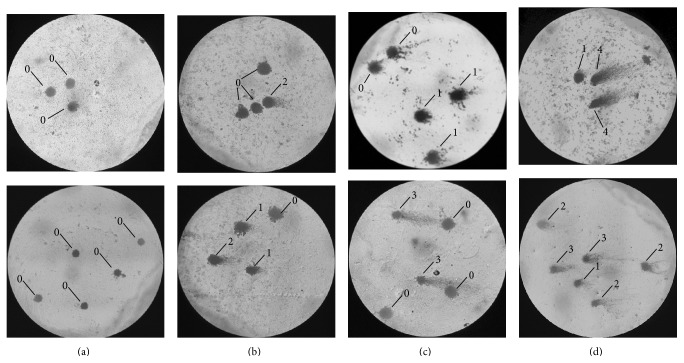
Photomicrograph of the comet test indicative of the types of damage. (a) Control group (CG); (b) beginning of treatment (C0); (c) 21 days after second cycle of chemotherapy (C2); (d) 21 days after the fourth cycle (C4).

DNA in the tail was organized into five classes: (i) class 0: undamaged, with no tail; (ii) class 1: with tail shorter than the diameter of the head (nucleus); (iii) class 2: with tail length between one and two times the diameter of the head; (iv) class 3: with tail longer than two times the diameter of the head; and (v) class 4: comets with no heads [33] (Figure 2).

**Figure 3:**
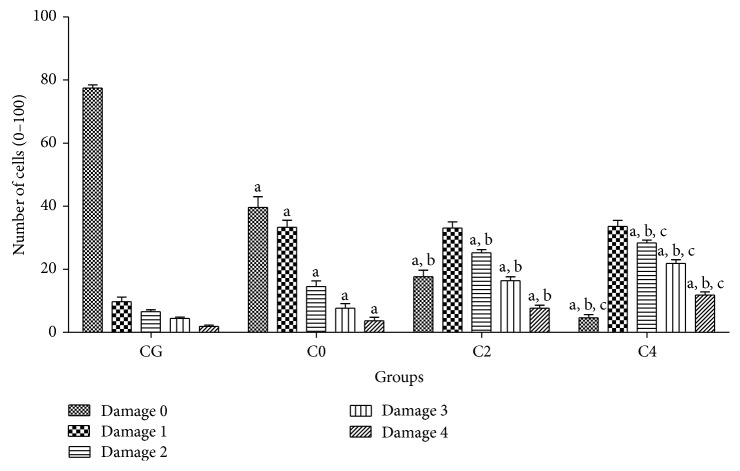
Profile of DNA damage in lymphocytes evaluated by the alkaline comet assay (single cell gel electrophoresis) for each phase of chemotherapy. Control group (CG) is represented by healthy patients. Values represent mean ± S.E.M. ^a^ *P* < 0.05 when compared with control group (CG) by ANOVA followed by *t-Student-Newman-Keuls*. ^b^ *P* < 0.05 when compared with C0 group (before chemotherapy) and ^c^ *P* < 0.05 when compared with C2 group (second cycle of chemotherapy).

With the application of alkaline comet assay it was possible to observe an increase (*P* < 0.05) in the classes of DNA damage in lymphocytes of patients with breast cancer (C0) in the control group. This condition is increased (*P* < 0.05) in C2 and C4 (Figure 3).

**Figure 4:**
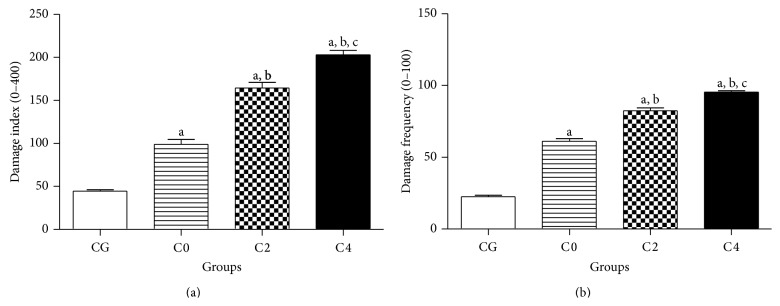
DNA damage investigation by the alkaline comet assay (single cell gel electrophoresis) carried out in lymphocytes of patients with breast cancer before (C0), during (C2), and after AC (C4) chemotherapy. Control group (CG) is represented by healthy patients. Analyses were performed by light microscopy at 100x magnification with immersion oil. Values represent mean ± S.E.M. ^a^ *P* < 0.05 when compared with control group (CG) by ANOVA followed by *t-Student-Newman-Keuls*. ^b^ *P* < 0.05 when compared with C0 group (before chemotherapy) and ^c^ *P* < 0.05 when compared with C2 group (second cycle of chemotherapy).

DNA damage in lymphocytes of patients with breast cancer increased by 122.6% (98.89 ± 5.56) compared to the control group (44.43 ± 1.67). After AC chemotherapy, there was an increase of 66.25 and 105.2% of damage index in C2 (164.4 ± 6.36) and C4 groups (202.9 ± 5.34) in comparison with C0 group (98.89 ± 5.56). In a similar way, an increase of 23.2% after the fourth cycle was noted when compared to the C2 group (Figure 4(a)).

An increase of 171.1% in frequency of DNA damage before chemotherapy (C0) (61.00 ± 2.01) was observed when compared to the CG (22.50 ± 0.94) (Figure 4(b)). AC chemotherapy raised this frequency by 34.9 and 56.3% in C2 (82.32 ± 2.08) and C4 (95.36 ± 0.99) groups, respectively, in comparison with the base* status* (C0). Similarly, in C4 patients, an increase of 15.8% was observed in comparison with the frequency of C2.

There was no correlation between sperm risk factors, age, smoking, and family history with the disease, as well as the levels of oxidative stress assessed by measurements of enzymes catalase, superoxide dismutase and glutathione peroxidase and malondialdehyde levels, nitrite, and reduced glutathione (*P* > 0.05). However, a significant positive correlation (correlation factor = 0.389 and *P* = 0.041) was observed between race and nitrite levels after chemotherapy (Q4) and a negative correlation was observed with nitrite levels (correlation factor = −0.474, *P* = 0.011) in the diagnosis and activity of superoxide dismutase (correlation factor = −0.389, *P* = 0.041) after chemotherapy (Q4) to marital status. There was also a negative correlation (correlation factor = −0.460, *P* = 0.014) between the practice of physical exercises and malondialdehyde levels during chemotherapy. No correlation was observed between ER and PR receptors with oxidative stress, except between HER2 and glutathione peroxidase in Q2 group with 0.412 correlation factor *P* = 0.29.

Regarding the genotoxicity and oxidative stress, positive correlations were observed for the contents of DNA damage assessed at diagnosis (QD) compared to those obtained during (Q2) and after (Q4) chemotherapy, with 0.663 correlation factor 0.537 and *P* = 0.000 and 0.003, respectively. A negative correlation was observed between levels of DNA damage during chemotherapy (Q2) and nitrite levels (Q4), as well as between frequency of damage (QD) and nitrite in group Q4 and Q2 catalase group.

## 4. Discussion

Breast cancer is the second most common cancer in women over the age of 50. It is often first detected as an abnormality on a mammogram before the patient or health care provider feels it. Early cases may be asymptomatic, and pain and discomfort are typically not present. Breast cancer can begin in different areas of the breast, such as ducts and lobules. Ductal carcinoma* in situ* (DCIS) is the most common noninvasive or preinvasive type with chances of a recurrence under 30% within 5–10 years after initial diagnosis. On the other hand, invasive ductal carcinoma (IDC), known as infiltrating ductal carcinoma, is the most common type of invasive breast cancer, representing around 80% of cases. About two-thirds of women are 55 or older when they are diagnosed with an IDC [34].

Many studies have reported that reactive oxygen species (ROS) and reactive nitrogen species (RNS) are involved in the etiology and progression of various cancers [35–38]. These reactive species have been associated with the development of carcinogenesis by activating diverse types of DNA damage, contributing to the emergence of mutations and chromosomal aberrations in the inflammatory process and leading to intense tissue disorganization and injuries [39].

An alternative method of analyzing oxidative stress is achieved by quantification of lipid peroxidation. The lipid radical is unstable and degrades very rapidly into secondary products. Most of them are electrophilic aldehydes, such as TBARS, which is the main marker of oxidative injury in the unsaturated lipids in cell membranes, leading to oxidation of fatty acids (LH) and formation of the lipid radical (L•) [40]. Therefore, TBARS is an important indicator of oxidative stress [16]. The present study demonstrated an elevation of TBARS levels in AC-treated breast cancer patients compared to controls, corroborating previous studies [1, 35] and suggesting severe lipid peroxidation. These changes may be attributed to the production of hydroxyl radicals, which participate directly in the lipid peroxidation process, inducing a disturbance in membrane structure [41].

The evaluation of nitrite concentration has been used as an index of endogenous NO production in biological systems in distinct pathological processes beyond its physiological properties such as vasodilation, neurotransmission, and immune response [3, 18]. It was noted that serum nitrite content determined by the Griess method increased in patients with breast cancer. Higher levels of nitrite and nitrate are related to inflammation caused by diseases and pharmacotherapies [39]. Interestingly, the results of present study showed that, even before the chemotherapy cycles, the disease itself induced NO_2_^−^ generation and revealed an increase according to the treatment when compared to the baseline. Previous findings found analogous results, exhibiting increases in lipid oxidation activated by NO_2_^−^ levels or nitric oxide [5, 37]. Nitric oxide has a dual role in tumor invasion and metastasis, inducing tumor growth or promoting tumoricidal activity [3]. Our results support the hypothesis that breast cancers are associated with increased nitric oxide levels whose changes are linked to inflammatory process [38]. Prior analyses in 14 patients with breast carcinomas showed no elevation of serum TBARS. However, increased NO concentrations were detected [42].

The extent of oxidative damage depends not only on ROS levels, but also on mechanisms of cellular antioxidant defenses. Low level of GSH, a molecule of critical importance in maintaining the stability of erythrocytes membranes, is related to cellular defense against xenobiotics and harmful compounds such as free radicals and hydroperoxides [43]. This drop in GSH was also observed in erythrocytes of the patients. An additional reduction in GSH levels was observed in healthy patients and those under chemotherapy. Glutathione acts as the first line of defense against free radicals produced by antitumor molecules. Decreased GSH levels can be explained by a decrease in GSH synthesis and/or increased consumption to remove peroxides and xenobiotics [44].

Metabolites generated by CMF (cyclophosphamide, methotrexate, and 5-fluorouracil) induced lipid peroxidation by inactivation of GSH levels and SOD, CAT, GPx, and GST activities in erythrocytes of patients with breast cancer, thereby rendering the system inefficient in management of the free radical attack. Acrolein and phosphoramide mustard are the metabolites of cyclophosphamide that are among the causative agents, which reduce the activity of SOD, CAT, GPX, glutathione-S-transferase, and glucose-6-phosphate dehydrogenase in erythrocytes of CMF treated breast cancer patients [45]. In the present study, GSH concentration and GPx activity were also observed just before AC chemotherapy. Our data demonstrate that GPx activity decreased, compared to the control group. However, this decrease was seen before the start of chemotherapy, suggesting no change in the activity of this enzyme for the therapeutic protocols used, since the reductions for during and after chemotherapy evaluation were similar to those observed prior to chemotherapy. Furthermore, these results suggest that the establishment of the pathophysiology of breast cancer may be a compromise in the activity of this enzyme. Present results and outcomes of Singh et al. [46] and Prabasheela et al. [47] also revealed, during chemotherapy FAC (5-fluorouracil, doxorubicin, and cyclophosphamide) or AC, a decrease in the nonenzymatic antioxidant GSH levels in patients with breast cancer before chemotherapy. On the other hand, additional studies did not find decrease in GPx activity before or after administration of chemotherapeutics [47, 48].

In relation to the CAT levels, our findings did not show differences before and during chemotherapy, presenting only increasing activity after treatment. On the other hand, while some studies found increases only after chemotherapy [48, 49], others observed decreases in CAT activity before and after chemotherapy [5, 40]. Since antioxidants can activate gene expression via the antioxidant response element [50], overexpression of enzymatic activities can explain these findings [40]. Similarly, SOD activity was elevated in patients with breast cancer before, during, and after chemotherapy. Hasan et al. [51] also showed plasma SOD activity increasing in patients with breast carcinoma compared to patients with benign tumors, suggesting that elevated total SOD might reflect a response to oxidative stress and then may predict a state of excess reactive oxygen species in the carcinogenesis process. Analogous outcomes were described by Badid et al. [52] before chemotherapy in erythrocytes of 38 patients with ductal breast cancer. Nevertheless, Gupta et al. [53] found a decrease in SOD activity in serum from 30 women. Patients with breast cancer in chemotherapy with epirubicin (90 mg/m^2^) and cyclophosphamide (600 mg/m^2^) also showed reduced CAT, SOD, GSH, and GPx activity and increased TBARS levels [54]. Some of these parameters are contradictory when compared to the outcomes in the present study. These differences are probably explained by the fact that enzymatic activity of antioxidant defenses is more expressed at the cytoplasmic and mitochondrial cellular level, especially for SOD [46].

In this study, the genotoxic profile assay of the patients with breast cancer under treatment with AC was also investigated. This evaluation was carried out by alkaline comet assay, a well-established, simple, versatile, rapid, visual, and sensitive tool used to assess DNA damage and repair quantitatively as well as qualitatively in individual cell populations [55]. Some other forms of DNA damage such as DNA cross-links (e.g., thymidine dimers) and oxidative DNA damage may also be assessed using lesion-specific antibodies or specific DNA repair enzymes in the comet assay. This technique has gained wide acceptance as a valuable tool in fundamental DNA damage and repair studies, genotoxicity testing, and human biomonitoring [56]. Relative to other genotoxicity tests, such as chromosomal aberrations, sister chromatid exchanges, alkaline elution, and micronucleus assay, the advantages of the comet assay include its demonstrated sensitivity for detecting low levels of DNA damage (one break per 10^10^ Da of DNA) [57].

The pathological condition significantly raised the damage indices and frequencies in lymphocytes when compared with the normal control group, confirming previous investigations performed by Sánchez-Suárez et al. [6] and Agnoletto et al. [4]. These effects on DNA structure remained elevated up to 80 days after the end of exposure to FEC (5-fluorouracil, epirubicin, and cyclophosphamide) [6]. As seen in this work, Vaghef et al. [9] showed significant increase in DNA damage on lymphocytes of patients treated with cyclophosphamide.

In fact, antineoplastic agents are currently used in clinical studies which induce breaks in mammalian DNA strands as seen with topoisomerase I (camptothecin) and topoisomerase II (etoposide) inhibitors and 5-FU [58]. This, for example, is an antimetabolite widely used to treat breast adenocarcinoma and cancers of the gastrointestinal tract, head, and neck due to its inhibitory action on the enzyme thymidylate synthase, among other mechanisms, despite their* in vivo* clastogenic activity [59, 60]. Moreover, doxorubicin, beyond inhibiting topoisomerase II, also induces apoptosis and free radical formation [10, 45, 61]. These can cause DNA adducts, cross-links, double strand breaks, and single strand breaks. So, any biological reaction has potentiality to induce carcinogenesis [6, 62].

## 5. Conclusion

Patients with breast cancer under chemotherapy presented antioxidant status indicative of oxidative stress before, during, and after chemotherapy, as well as increasing genotoxic damage in all stages of the treatment. These results highlight the importance of monitoring patients in chemotherapy, especially using cytogenetic and molecular markers in order to provide new prognostic findings to the treatment as a strategy to reduce recurrences and to improve quality of life.

## Figures and Tables

**Table 1:** Demographic, medical, and lifestyle data of the patients.

Characteristics	Control group	Breast cancer
Total of patients	28 (100.0)	28 (100.0)
Age in years (mean ± SD)	48.1 ± 11.8	50.8 ± 12.8
Race [*n* (%)]		
Caucasian	20 (71.4)	22 (78.6)
Non-Caucasian	8 (28.6)	6 (21.4)
Menopause [*n* (%)]		
Premenopausal	19 (67.9)	20 (71.4)
Postmenopausal	9 (32.1)	8 (28.6)
Family history of breast cancer [*n* (%)]		
Yes	6 (21.4)	15 (53.6)
No	22 (78.6)	13 (46.4)
Physical exercises [*n* (%)]		
Yes	11 (39.3)	9 (32.1)
No	17 (60.7)	19 (67.9)
Smoker [*n* (%)]		
Never smoked	20 (71.4)	12 (42.9)
Ex-smoker	8 (28.6)	13 (46.4)
Smoking	0 (0.0)	3 (10.7)
Marital status [*n* (%)]		
Single	7 (25.0)	6 (21.4)
Married	16 (57.1)	15 (53.6)
Divorced	1 (3.6)	3 (10.7)
Widow	4 (14.3)	4 (14.3)

SD: standard deviation.

**Table 2:** Clinical characteristics of patients with breast ductal carcinoma (*n* = 28).

Characteristics	Breast cancer
Cancer sites [*n* (%)]	
Left breast	11 (39.3)
Right mama	17 (60.7)
Clinical stage [*n* (%)]	
Grade 1	4 (14.3)
Grade 2	10 (35.7)
Grade 3	14 (50.0)
Estrogen receptor [*n* (%)]	
Negative	7 (25.0)
Positive	21 (75.0)
Progesterone receptor [*n* (%)]	
Negative	7 (25.0)
Positive	21 (75.0)
HER2/neu [*n* (%)]	
Score 0	9 (32.1)
Score +1	10 (35.7)
Score +2	1 (3.6)
Score +3	8 (28.3)

HER2/neu: human epidermal growth factor receptor 2.

**Table 3:** Biomarkers levels of oxidative stress in antioxidant enzymatic system of patients with breast cancer before (C0), during (C2), and after chemotherapy (C4) and control group.

Groups	TBARS levels (nM/mg de protein)	NO_2_^−^ content (*µ*M/mg protein)	GSH concentration (U/g protein)
Control	0.37 ± 0.09	0.16 ± 0.05	36.13 ± 7.65
C0	1.42 ± 0.45^a^	1.16 ± 0.62^a^	24.94 ± 1.51^a^
C2	4.76 ± 0.68^a,b^	1.81 ± 0.02^a,b^	19.30 ± 0.74^a,b^
C4	11.98 ± 0.65^a.b,c^	3.49 ± 0.07^a,b,c^	17.75 ± 0.46^a,b^

TBARS: thiobarbituric acid reactive substances levels, NO_2_^−^: nitrite content, and GSH: reduced glutathione concentration. Values represent mean ± S.E.M. ^a^ *P* < 0.05 when compared with control group (CG) by ANOVA followed by *t*-Student-Newman-Keuls. ^b^ *P* < 0.05 when compared with C0 group (before chemotherapy) and ^c^ *P* < 0.05 when compared with C2 group (second cycle of chemotherapy).
